# The Role of Entrustable Professionals Activities in the Training of Specialists in Gynecology and Obstetrics

**DOI:** 10.1055/s-0042-1755339

**Published:** 2022-10-10

**Authors:** Gustavo Salata Romão

**Affiliations:** 1 Medical Course, Universidade de Ribeirão Preto, Ribeirão Preto, São Paulo, Brazil.

Competency-based training emerged in the early 2000s and stands out as the primary internationally recognized guidance model for undergraduate and postgraduate (PG) training. 1 The knowledge, skills, and attitudes expected from physicians and specialists were established in competencies frameworks. In Brazil, the Medical Residency Commission (COREME) of the Brazilian Federation of Gynecology and Obstetrics Associations (Febrasgo) led the development and validation of the Competency Framework in Gynecology and Obstetrics, 2 and subspecialties of Fetal Medicine, Human Reproduction, Sexology, and Gynecological Endoscopy. 1

However, the many subcomponents described in the Frameworks make it impossible for preceptors and supervisors to assess them throughout PG training. 3

To facilitate training and assessment of PG trainees, these competencies, which are attributes of an individual, had to be transformed into clearly observable and verifiable activities by preceptors and supervisors. 4 In 2007, these activities were identified as Entrustable Professional Activities (EPAs). 4

An EPA is defined as “a unit of professional practice that can be fully entrusted to an apprentice (PG trainee or medical student) when he demonstrates the necessary competencies to perform it independently and without supervision.” 1 A unit of practice can refer to a single task or activity (such as clinical examination of a pregnant woman or repair of a perineal tear) or to a set of tasks or activities (such as antenatal care or care for normal-risk delivery) undertaken by the apprentice physician that can be assessed by their preceptor or supervisor. Proficiency in performing each EPA, or unit of practice, becomes a prerequisite to authorize the apprentice or PG trainee to undertake it autonomously or independently of supervision, which is a process called entrustment. 1

The competencies required to perform each EPA do not need to be assessed individually. Entrustment in each activity presupposes the acquisition of knowledge, skills, and attitudes described in the Competency Framework, which enables and facilitates the PG trainee assessment by preceptors and supervisors. 5

In 2021, Febrasgo developed the Brazilian EPAs in Gynecology and Obstetrics at a national level, and this work was led by COREME. The chosen methodology was the Panel of Experts, as established in the literature and used in the development of the Competency Framework in Gynecology and Obstetrics. 2 6

Based on national and international references, 2 7 8 9 COREME/Febrasgo defined and validated 21 EPAs for the specialty of Gynecology and Obstetrics in Brazil. This comprehensive and detailed work involved more than 250 experts in different specialty areas. Therefore, the Gynecology and Obstetrics EPAs should guide PG trainees' entrustment in this specialty. The titles chosen for the 21 EPAS were: Promoting Prenatal Care for Low-Risk Pregnancies, Promoting Prenatal Care for High-Risk Pregnancies, Promoting Birth Care for Low-Risk Pregnancies, Promoting Birth Care for High-Risk Pregnancies, Promoting Postpartum Care, Promoting Care in Obstetric Emergencies, Promoting Care in Gynecological Emergencies, Promoting Care in Infectious Diseases, Promoting Care in Premalignant Genital Tract Lesions, Promoting Care in Gynecologic Oncology, Promoting Care in Mastology, Promoting Care in Pelvic Floor Disorders, Promoting Care in Chronic Pelvic Pain, Promoting Care in Abnormal Uterine Bleeding, Promoting Care in Contraception and Reproductive Planning, Promoting Care in Gynecologic Endocrinology, Promoting Care for the Infertile Couple, Promoting Care in Child and Pubertal Gynecology, Promoting Care in Climacteric and Senescence, Promoting Care in Sexual Dysfunctions, and Promoting Care in Violence Against Women. 10

The entrustment of PG trainees in performing EPAs is essential to ensure patients' safety, as it restrains autonomous medical activities and procedures only to the qualified ones. Therefore, it prevents trainees who have not yet acquired proficiency in these activities from performing care or decisions for which they are still not adequately prepared. 1 4 5

Through the development and validation of EPAs, Febrasgo enhances medical education and opens new perspectives to qualify the training of professionals who will care for women's health in Brazil.
